# The Effect of Multilevel Carbon Reinforcements on the Fire Performance, Conductivity, and Mechanical Properties of Epoxy Composites

**DOI:** 10.3390/polym11020303

**Published:** 2019-02-12

**Authors:** Andrea Toldy, Gábor Szebényi, Kolos Molnár, Levente Ferenc Tóth, Balázs Magyar, Viktor Hliva, Tibor Czigány, Beáta Szolnoki

**Affiliations:** 1 Department of Polymer Engineering, Faculty of Mechanical Engineering, Budapest University of Technology and Economics, Műegyetem rkp. 3-9., H-1111 Budapest, Hungary; szebenyi@pt.bme.hu (G.S.); molnar@pt.bme.hu (K.M.); tothl@pt.bme.hu (L.F.T.); magyarb@pt.bme.hu (B.M.); hlivav@pt.bme.hu (V.H.); czigany@pt.bme.hu (T.C.); 2 MTA–BME Research Group for Composite Science and Technology, Műegyetem rkp. 3., H-1111 Budapest, Hungary; 3 Soete Laboratory, Department of Electrical Energy, Metals, Mechanical Constructions and Systems, Faculty of Engineering and Architecture, Ghent University, Technologiepark 903., B-9052 Zwijnaarde, Belgium; 4 Department of Organic Chemistry and Technology, Faculty of Chemical Technology and Biotechnology, Budapest University of Technology and Economics, Budafoki út 8, H-1111 Budapest, Hungary; bszolnoki@mail.bme.hu

**Keywords:** carbon nanotube, carbon nanofiber, flame retardancy, thermal conductivity, carbon fiber reinforced epoxy composite

## Abstract

We studied the effect of a multilevel presence of carbon-based reinforcements—a combination of conventional load-bearing unidirectional carbon fiber (CF) with multiwalled carbon nanotubes (CNT) and conductive CNT-containing nonwoven carbon nanofabric (CNF(CNT))—on the fire performance, thermal conductivity, and mechanical properties of reference and flame-retarded epoxy resin (EP) composites. The inclusion of carbon fibers and flame retardant reduced the peak heat release rate (pHRR) of the epoxy resins. The extent to which the nanoreinforcements reduced the pHRR depended on their influence on thermal conductivity. Specifically, high thermal conductivity is advantageous at the early stages of degradation, but after ignition it may lead to more intensive degradation and a higher pHRR; especially in the reference samples without flame retardant. The lowest pHRR (130 kW/m^2^) and self-extinguishing V-0 UL-94 rating was achieved in the flame-retarded composite containing all three levels of carbon reinforcement (EP + CNF(CNT) + CNT + CF FR). The plasticizing effect of the liquid flame retardant impaired both the tensile and flexural properties; however, it significantly enhanced the impact resistance of the epoxy resin and its composites.

## 1. Introduction

The discovery and application of nanosized reinforcing materials has fundamentally changed the research of polymer composites. Currently, carbon-based nanoreinforcements are in the centre of interest, due to their unique mechanical properties and the abundant forms available [1]. The effects of carbon-based nanosized reinforcements in polymer composites are widely studied—as besides the mechanical enhancement, other characteristics, such as gas barrier properties, electric and thermal conductivity, and flame retardancy can be simultaneously improved without further additives [2,3]. Recently, Wang et al. [4] reviewed the preparation and properties of multifunctional polymer composites with carbon-based materials (including graphite, graphene, carbon nanotubes, and fullerenes) and highlighted the fire performance of these additives. In this research area, most of the relevant publications focus on the application of nanosized carbon reinforcement on the surface of fiber-reinforced polymer composites in order to improve their fire performance.

Wu et al. [5] covered the surface of polyhedral oligomeric silsesquioxanes (POSS)/glass fiber composites with a carbon nanotube (CNT) membrane (buckypaper), prepared by the filtration of a CNT suspension, in order to improve the flame retardancy of the composites. According to their results, the buckypaper acted as a thermally stable physical barrier and reduced the heat release rate (HRR), the peak heat release rate (pHRR), and smoke production.

Wu et al. [6] also applied single-walled carbon nanotube (SWCNT) and multiwalled carbon nanotube (MWCNT) membranes (buckypaper) and carbon nanofiber (CNF) paper to the surface of carbon fiber reinforced epoxy resin composites and investigated their effect on flammability by cone calorimetry. According to their results, SWCNT buckypaper and CNF paper did not show a notable improvement in fire retardancy, while MWCNT-based buckypaper acted as an effective flame-retardant shield and reduced the peak heat release rate by more than 60%. They explained the different behavior of the buckypapers by the better thermal stability of the MWCNT buckypaper and its smaller pore size, which led to a lower gas permeability than in the case of the CNF paper.

Fu et al. [7] applied a mixed SWCNT and MWCNT membrane to the surface of polyimide/carbon fiber composites. They compared the effect of direct mixing 5 wt % CNTs into the matrix material to the use of CNT buckypaper, concluding that although the decrease in the pHRR was similar (38% vs. 40%), the CNT buckypaper reduced the total heat release rate far more efficiently (26% vs. 3.7%).

Wu et al. [8] applied SWCNT and MWCNT membranes to the surface of epoxy and bismaleimide (BMI) carbon fiber composites. They explained the reduction in the pHRR by 60% and in smoke generation by 50% with the high thermo-oxidative stability of the buckypapers, which made them an excellent fire-retardant shield.

Zhuge et al. [9] modelled the thermal degradation of glass fiber reinforced polyester composites coated with CNF-based nanopapers which were subjected to different heat fluxes. The one-dimensional transient finite difference model they developed numerically demonstrated that the nanopaper coating helped to retain the structural integrity of the composite, as it reduced its mass loss and cold side temperature and eventually improved the mechanical properties of the composites.

In our previous paper [10], we investigated the effects of a multilevel presence of nanosized carbon reinforcements (CNT embedded in CNF and in the matrix itself) on the electrical and thermal conductivity, as well as on the mechanical properties, of epoxy resin composites for aircraft applications. The incorporation of CNTs into the carbon nanofibers prepared by electrospinning leads to a significant increase in both electrical and thermal conductivity, compared to conventional composites. This suggests a cost-effective and weight-saving way to substitute the metal meshes currently applied for the lightning protection of structural composite aircraft wing and fuselage shells [11]. As the nanofibers themselves do not provide enough reinforcement, the combination of conventional load-bearing unidirectional carbon fiber reinforcement with conductive nonwoven carbon nanofabric seems a more realistic approach to reach industrial applicability. While electric conductivity is essential to disperse electric charge on the surface of the composite, the increase in thermal conductivity not only dissipates the immense heat produced by an accidental lightning strike but also affects the fire performance of the composites [12,13]. When metal components are replaced with fiber-reinforced polymer composites in aircraft structures, due to their outstanding mechanical properties, low weight, and appropriate fire retardancy [14], reasonable electrical and heat conductivity become essential feature. Therefore, the purpose of the current research project was to investigate the effect of multilevel carbon-based reinforcements on the fire performance, conductivity, and mechanical properties of epoxy resin composites, both alone and in combination with a traditional flame retardant.

## 2. Materials and Methods

### 2.1. Preparation of Carbon Nanofiber Mats

**Figure 1:**
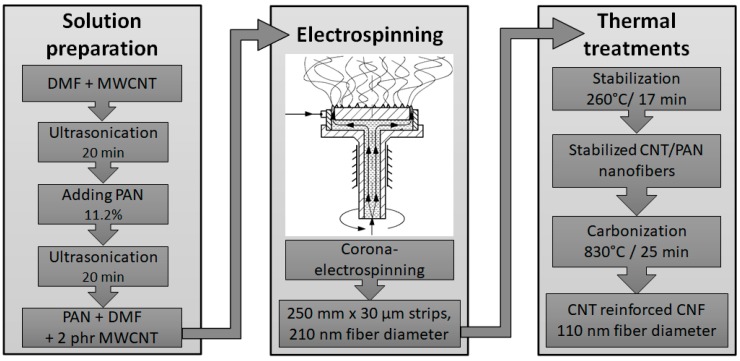
Preparation process of carbon nanofiber mats. DMF: dimethylformamide; MWCNT: multiwalled carbon nanotube; CNT: carbon nanotube; PAN: polyacrylonitrile; CNF: carbon nanofiber.

The fabrication of CNT-containing polyacrylonitrile (PAN)-based nanofiber mats and their stabilization and carbonization leading to carbon nanofiber mats is discussed in detail in a preceding article of the authors [10]. The materials and process parameters are summarized here only in brief (Figure 1).

First, the solution for electrospinning was prepared: Bayer Baytubes C 150 HP multiwalled CNTs were added to dimethylformamide (DMF) (Molar Chemicals, Hungary) and sonicated for 20 min using a Bandelin Sonoplus HD 2200 ultrasonic homogenizer equipped with a UW 2200 ultrasonic converter (Bandelin, Germany). After that, the PAN copolymer grade for carbon fiber production (the exact composition was not disclosed by the manufacturer) was mixed with the dispersion and ultrasonic treatment was carried out again. The prepared solution contained 11.3 wt % PAN and 2 wt % of CNT relative to PAN.

Electrospinning was carried out using corona electrospinning method [15]. At corona electrospinning, the solution is supplied through an annular orifice and electrospinning jets are formed along the annulus, bounded by a sharp circular electrode from the outside. Other needleless electrospinning methods [16,17,18] use a high open liquid surface, which is this way eliminated, and high productivity can be achieved at the same time. The applied voltage was set to 50 kV, and the distance between the grounded metal plate electrode and the spinneret was 120 mm. The nanofibers were collected in a continuous process on a 40 g/m^2^ polypropylene nonwoven substrate with a constant traction speed of 10 cm/min. Subsequently, the obtained 25 cm wide and 20–30 μm thick PAN nanofabrics were peeled off from the substrate with ease. After nanofiber generation these samples were stabilized and then carbonized. The stabilization was accomplished in a Nabertherm furnace (Germany) under air at 260 °C for 17 min. The stabilized PAN fabric was carbonized under a N_2_ atmosphere at 830 °C. The temperature profile of the four zones of the BTU (UK) tunnel furnace was set to 200, 650, 830, and 200 °C. The total carbonization time of a sample was 25 min, which was set by the conveyor speed of the furnace.

### 2.2. Sample Preparation

An Ipox MR-3012 low viscosity glycerol-based aliphatic epoxy resin with an Ipox MH-3111 anhydride-type hardener (Budapest, Hungary) was used as the matrix material in a 100:116 mass ratio. A unidirectional carbon weave (Zoltek (Nyergesújfalu, Hungary) PX35 FBUD0300), acting as a conventional fibrous reinforcement, was applied, while multiwalled carbon nanotubes (CNTs) (Baytubes C150HP produced by Bayer MaterialScience A.G. (Leverkusen, Germany)), acting as nanosized reinforcements, were applied in the matrix. The CNTs were dispersed in the matrix by the masterbatch technology previously developed by the authors [19,20]. First, an 8% CNT-containing masterbatch was prepared on an Enrico Molteni CIEM (Senago (MI), Italy) three roll mill, which was diluted to 0.3% CNT content with MR-3012 resin by mixing in an overhead stirrer for 4 h. As a flame retardant (FR), resorcinol bis(diphenylphosphate) (RDP) (ICL Industrial Products (Beer Sheva, Israel); trade name: Fyrolflex RDP; P-content: 10.7%) was applied, and the phosphorus (P)-content of the matrix was 2.5% in each flame-retarded specimen.

Resin specimens were cast into silicone molds, while composites were produced in a one-sided sheet metal mold by using the hand lay-up method followed by vacuum pressing in a vacuum bag. For the composite preparation, 6 and 12 layers of conventional unidirectional carbon fiber (CF) fabric were used, respectively, which were interleaved in hybrid samples with 5 and 11 layers of CNF mat, then, in addition to the interlaminar layers, CNF mats were placed both on the top and on the bottom of the composites. The interleaves do not increase the composite thickness significantly, as the nanofibers penetrate between the conventional carbon fibers when applying compression during composite manufacturing [21]. This way 2 mm thick laminates were obtained for mechanical testing and 4 mm thick ones for the conductivity and fire tests. Both the resin and composite samples were cured under a vacuum in a Heraeus (Hanau, Germany) UT-20 oven at 80 °C for 4 h. The specimens were cut from the laminates using a Mutronic (Rieden, Germany) Diadisc diamond disc cutter to the desired specimen size. The compositions of the prepared samples are given in Table 1.

### 2.3. Characterization of the Fire Behavior

The fire performance of the reference and flame-retarded composites was characterized by limiting oxygen index measurements (LOI), according to the American Society for Testing and Materials (ASTM) D2863 method.

The LOI value expresses the lowest volume fraction of oxygen in a mixture of oxygen and nitrogen that supports the flaming combustion of a material that is initially at room temperature. A higher value indicates a less flammable material. The sample size was 120 mm × 15 mm × 4 mm.

Standard UL-94 flammability tests (according to ASTM D3081 and ASTM D635, respectively) were also carried out in order to classify the samples based on their ignitability, dripping, and flame-spreading rates and burning times in horizontal and vertical test setups. The sample size was 120 mm × 15 mm × 4 mm. The increasing values of the UL-94 ratings are as follows: HB, V-2, V-1, and V-0.

Mass loss type cone calorimeter tests were carried out by an instrument made by FTT Inc. (East Grinstead, UK), using the ISO 13927 standard method. Specimens (100 mm × 100 mm × 4 mm) were placed beneath the truncated cone-shaped radiant electrical heater of the instrument and were then exposed to a constant heat flux of 50 kW/m^2^ and ignited. Heat release values and mass reduction were continuously recorded during burning.

### 2.4. Thermal Conductivity Testing

The thermal conductivity measurements were performed in a guarded hot-plate setup [10]. The sample was placed between two 80 mm × 80 mm copper plates, one of which was heated to a given temperature. The temperatures were monitored by 2-2 built-in NTC thermistors (Epcos B57045K) inside both the heated and the cooled plate. The heating energy input was monitored by measuring the power consumption of the heating, and the losses were compensated by previously performed calibration measurements. The plates and the sample were placed in an isolated chamber. The thermal conductivity of the sample (*λ*) can be calculated by Equation (1) based on Fourier’s law from the sample thickness (*L_m_*), the heat flux passing through it (*P*—heating power), the surface of the specimen (*A*) perpendicular to the heat flux, and the temperature difference between the two sides of the specimen (*ΔT_m_*):

*λ* = *P*/(2*A*)*L_m_/*ΔT_m_* (1)

In all cases, the upper plate was tempered to 50 °C and the lower plate was heated with constant power. Each specimen was covered with thermally conductive silicone grease to minimize thermal contact resistance caused by the surface roughness of the plates and the specimen. The steady state was achieved in all measurements by applying a dwell time of 30 min to reach equilibrium. After reaching equilibrium, the measurement was conducted for 5 min with a data sampling rate of 1 Hz, and then the average was calculated.

### 2.5. Mechanical Testing

All mechanical tests were carried out on 80 mm × 10 mm × 2 mm oblong specimens.

Tensile measurements were performed according to EN ISO 527-2 using a Zwick (Ulm, Germany) Z250 computer-controlled tensile tester (equipped with a 5 kN load cell for resin testing and a 20 kN load cell for fiber-reinforced composites) with self-aligning grips at a 1 mm/min test speed and 40 mm initial grip distance on 5-5 specimens of each sample type. The fine strain measurement for the modulus calculation was performed using a Sobriety (Kuřim, Czech Republic) Mercury Monet DIC optical measurement system.

Three-point bending measurements were performed according to EN ISO 178/EN ISO 14125 on specimens using the same Zwick machine with a standard three-point bending fixture at a 1 mm/min test speed and 32 mm support span on 5-5 specimens from each sample type.

Charpy notched impact tests were carried out according to EN ISO 179-2 on specimens with a 2 mm deep standard machined notch (type A) in the centre. For the tests, a Ceast (Pianezza, Turin, Italy) Resil Impactor Junior instrumented pendulum equipped with a 2 J hammer was used on 7-7 specimens from each sample and a 2.9 m/s impact velocity and 62 mm support span were set. The force-time curves were registered by a Ceast DAS 8000 data acquisition unit.

Reference and flame-retarded (FR) epoxy resin (EP) composites reinforced with carbon nanotubes (CNT) and/or CNT-filled electrospun carbon nanofiber (CNF(CNT)) mats were prepared and their properties were compared to those of the neat epoxy resin matrix and conventional carbon fiber reinforced composites as well.

## 3. Results and Discussion

### 3.1. Fire Performance

The fire performance of the prepared samples was determined by means of the LOI and UL-94 tests and the mass loss type cone calorimetry measurements. The results are summarized in Table 2 and Table 3.

**Figure 2:**
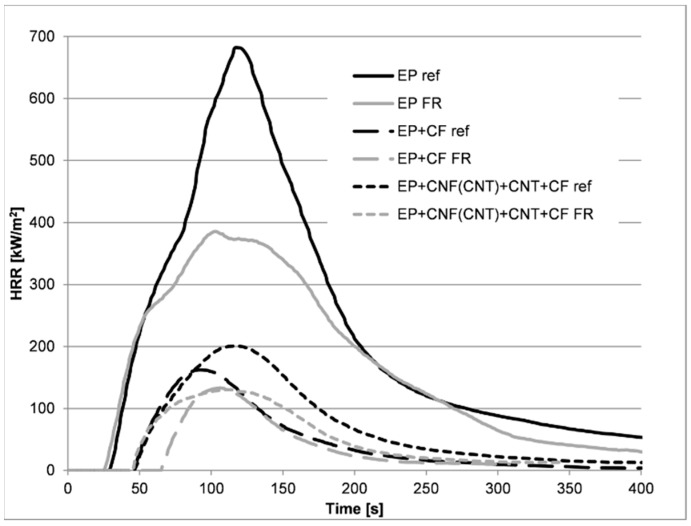
Heat release rate of reference and flame-retarded EP matrix and hybrid composite samples.

Concerning the mass loss type cone calorimetry measurements, it can be clearly seen that, with the addition of carbon fiber reinforcement, the time to ignition increased significantly both in the reference and the flame-retarded samples. The ignition of the FR composites occurred at around one minute, which meant that the time to ignition was almost three times longer than in the unreinforced reference EP matrix sample. This can be attributed primarily to the decreased amount of burnable matrix. It is also due to the good heat conductivity of the carbon fibers, which can lead the heat away from the sample surface towards the sample holder and thus delay the degradation of the polymer matrix [22]. The effect of the carbon fibers can also be seen in the peak heat release rate values of the reference and flame-retarded samples, which decreased drastically from 680 to 162 kW/m^2^ and from 386 to 133 kW/m^2^, respectively, solely with the addition of carbon fibers to the matrix (Figure 2). With the addition of CNTs to the matrix samples, the time of the pHRR increased, while no significant change in the pHRR values was detected. The effect of the addition of flame retardant was pronounced in the epoxy matrix materials without CF reinforcement—a decrease of about 40–45% was observed in the pHRR and the total heat release (THR) values. The fiber-reinforced samples had already decreased pHRR values compared to the matrices, which could only be slightly further decreased with the addition of FRs—the average heat release rate of around 165 kW/m^2^ decreased to around 135 kW/m^2^.

In Figure 2, the heat release curves of selected reference and FR samples can be seen. In the case of the carbon fiber reinforced composite reference samples, due to the addition of the nanoreinforcements (CNFs and CNTs), the pHRR value increased from 162 to 201 kW/m^2^. However, among the samples flame retarded by RDP, which acts mainly in the gas phase as a radical scavenger [23,24], the lowest pHRR was detected in the sample containing both nanoreinforcements (Table 2). To find an explanation for this different behavior, we compared the thermal conductivity of the composite samples (see Section 3.2.), and we found that both among the reference and flame-retarded samples, the one with the highest thermal conductivity had the highest pHRR, while the one with the lowest thermal conductivity had the lowest pHRR. Accordingly, a reinforcement with a high thermal conductivity is advantageous in the early stages of degradation, because it leads the heat away from the surface of the composite, thus lowering its temperature; however, after ignition the high conductivity of the composite may lead to more intensive degradation. The increase in the pHRR was more pronounced in the reference (1–6) samples, however, due to the overwhelming effect of the FR, the effect was less significant in the flame-retarded ones (7–12).

The addition of FR to the matrix increased the LOI from 23 to 32 *V*/*V*%, while the incorporation of the carbon fiber reinforcement led to a LOI of 33 *V*/*V*% in the reference sample and a LOI of 42 *V*/*V*% in the flame retarded one due to the decrease of the burnable matrix material. The addition of nanoscale reinforcements (CNTs or CNFs) to composites slightly decreased these LOI values, however, due to the FR, the LOI of the flame-retarded composites remained as high as 39 and 40 *V*/*V*% (Table 3).

Concerning the UL-94 results, it can be seen that the flame-spreading rate of the reference matrix increased with the addition of CNTs to the matrix. The addition of either carbon fibers or FR stopped horizontal burning, but neither was enough to prevent the samples from burning up to the holding clamp, thus almost all the samples have a HB rating. The addition of carbon nanofibers (CNF(CNT)) to the flame-retarded composites efficiently reduced the flaming of the samples, reaching a V-1 rating, however, the addition of CNTs to the CNF-containing FR composite was necessary to reach a V-0 rating in the UL-94 test (Table 3).

### 3.2. Thermal Conductivity

**Figure 3:**
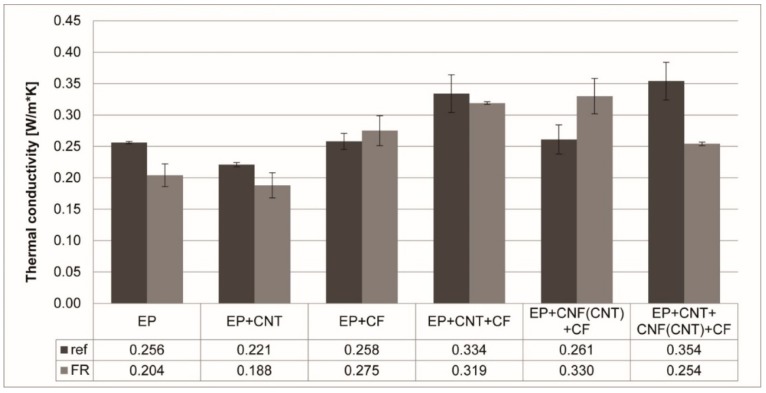
Thermal conductivity of the prepared reference (ref) and flame-retarded (FR) samples.

The results of the thermal conductivity measurements are summarized in Figure 3. The results represent the average values measured during 5 min after reaching an equilibrium state. 

By comparing the EP + CF reference and the EP + CNF(CNT) + CF reference samples, it can be concluded that the inclusion of CNF(CNT) increased the thermal conductivity only negligibly, and the inclusion of all three levels of carbon reinforcement (CF, CNT, and CNF(CNT)) was necessary to reach the highest thermal conductivity among the reference samples (EP + CNT + CNF(CNT) + CF reference). The addition of RDP to the matrix slightly lowered the thermal conductivity. Furthermore, it can be noted that, in all samples containing CNTs, the flame-retarded versions exhibited a lower thermal conductivity than their reference counterparts; however, the trend reversed for the samples reinforced only with CF or the combination of CNF(CNT) and CF, and the flame-retarded versions had a higher thermal conductivity. This difference may be attributed to the polar nature of the flame retardant, namely, the dispersion of apolar CNTs in a more polar flame-retarded matrix may be less efficient than in the reference epoxy matrix, resulting in the lower conductivity of the CNT-containing FR composites.

### 3.3. Mechanical Properties

The mechanical characterization of the composites was carried out through static tensile and three-point flexural tests and edgewise Charpy impact tests. While the tensile tests resemble pure tension along the direction of the reinforcing fibers, so the characteristics are dominated by the reinforcement, the three-point bending and edgewise Charpy impact tests both provide information on the interlaminar properties.

**Figure 4:**
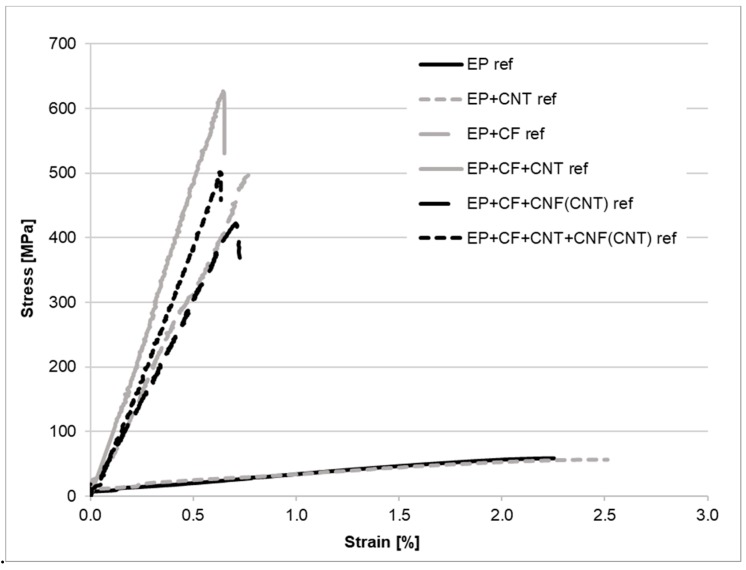
Tensile curves of the reference (ref) samples.

**Figure 5:**
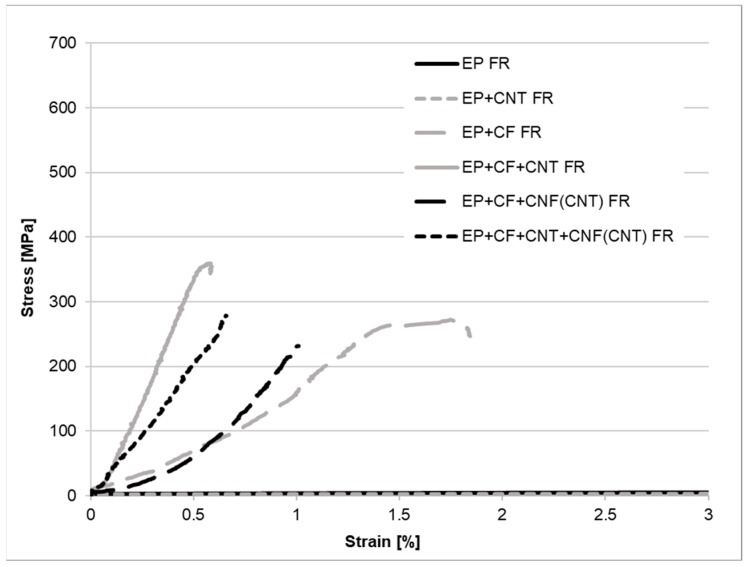
Tensile curves of the flame-retarded (FR) samples.

The results of the tensile tests are presented in Table 4 and in Figure 4 and Figure 5. If we compare firstly the resin specimens without CF reinforcement, clear tendencies can be observed. The incorporated CNTs behave mostly as a filler, because of their moderate interfacial adhesion towards the matrix, causing a significant decrease in the tensile strength and an increase in the modulus. The decrease of the tensile strength may have been caused by the crack-initiating behavior of the CNT aggregates as well [19,25], although no aggregates were detectable in the matrix samples with scanning electron microscopy at 10,000× magnification. However, the presence of smaller CNT-aggregates can be presumed on the basis of the significantly increased standard deviation of the measured values. The added flame retardants noticeably lowered both the strength and modulus and increased the elongation at break of the resin, probably by the plasticizing effect of the liquid flame retardant proved in the previous work of the authors [23,24,26]. This was also the case when applying the CNTs and the FR simultaneously. When comparing the samples with long fiber reinforcement, the CFs can compensate for the weak matrix properties in the flame-retarded resin. When comparing the results of the composites containing CNF interlayers, it can be seen that the composite properties are not modified when no FR additives are present, but, when the resin is flame-retarded, noteworthy drawbacks in the tensile properties can be observed, probably because the load transfer between the CF layers is hindered by the CNF interlayers containing a weak, plasticized matrix.

The results of the three-point flexural tests are presented in Table 4. Here the main tendencies are similar to the ones observed in the tensile tests. In flexural loading of a reinforcing structure, significant interlaminar shear effects are also present, cracks also have to propagate in the interlayer, and this can explain the good results of the neat EP system reinforced with CNTs, CFs, and CNFs. The plasticizing effect of the FR additive in the resin caused a decrease both in the modulus and in the strength of the FR systems. During the tests, significant delamination was observed in samples 11 and 12.

**Figure 6:**
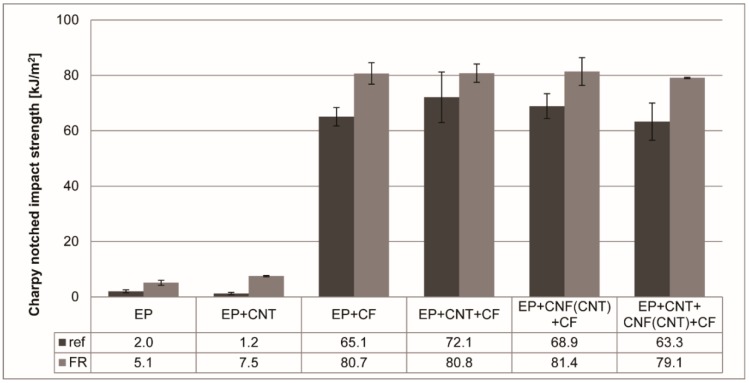
Charpy notched impact strength of the prepared reference (ref) and flame-retarded (FR) samples.

Nevertheless, the plasticization was beneficial to the impact properties (Figure 6)—all FR composites outperformed the neat matrix specimens or composites in energy consumption during the fracture process.

## 4. Conclusions

The main purpose of this research was to investigate the applicability of the interleaving concept [27] using nanoengineered carbon-based reinforcements in flame-retarded fiber-reinforced polymer composites. For this reason, we prepared reference and flame-retarded (FR) epoxy resin (EP) composites reinforced with conventional carbon fibers (CF), carbon nanotubes (CNT), and/or CNT-filled electrospun carbon nanofiber (CNF(CNT)) and compared their fire performance, thermal conductivity, and mechanical properties.

The carbon fiber reinforcement significantly increased the time to ignition and reduced the peak heat release rate (pHRR) both in the reference and flame-retarded samples, due to the decreased amount of burnable matrix and the good heat conductivity of the carbon fibers, which led the heat away from the sample surface. With the addition of CNTs to the matrix samples, the time of the pHRR increased, but no significant change in the pHRR was detected. The change in the pHRR caused by the addition of nanoreinforcements (CNFs and CNTs) to the composites was connected to the thermal conductivity of the composites—the composites with the highest thermal conductivity had the highest pHRR, while the composites with the lowest thermal conductivity had the lowest pHRR. Based on these results, we assume that a high thermal conductivity is advantageous in the early stages of degradation, but that after ignition it may lead to more intensive degradation, especially in the reference samples without flame retardant. The pHRR of the fiber-reinforced samples could be further decreased with the addition of a flame retardant (FR) from around 165 kW/m^2^ to around 135 kW/m^2^. The lowest pHRR (130 kW/m^2^) was achieved in the flame-retarded composite containing all three levels of carbon reinforcement (EP + CNF(CNT) + CNT + CF FR).

The addition of nanoscale reinforcements (CNTs or CNFs) to composites slightly decreased the LOI values, but due to the FR, the LOI of the flame-retarded composites remained as high as 39 and 40 *V*/*V*%. During the UL-94 tests, CNTs increased the flame-spreading rate of the reference matrix. However, the inclusion of carbon nanofibers (CNF) in flame-retarded composites reduced the flaming of the samples, resulting in a V-1 rating. For the self-extinguishing V-0 rating, the combined application of CNTs and CNFs was necessary.

The thermal conductivity of the matrix samples decreased with the addition of the RDP flame retardant. Furthermore, in all samples containing CNT, the flame-retarded versions had lower thermal conductivity than their reference counterparts, which may be explained by the less effective dispersion of the apolar CNTs in the more polar FR matrix.

The plasticizing effect of the liquid flame retardant decreased both the tensile and flexural strength and modulus of the epoxy resin and composite samples. The mechanical properties of the carbon fiber reinforced systems are sufficient for load-bearing structural composites, and the enhanced impact resistance is a positive addition, especially in the transport sector where impact loads are common during both normal operation and accidents.

## Figures and Tables

**Table 1:** Composition of the prepared reference and flame-retarded samples.

No.	Sample Code	Matrix	Reinforcement	Flame Retardant
1	EP ref	EP	-	-
2	EP + CNT ref	EP with 0.3% CNT	-	-
3	EP + CF ref	EP	CF	-
4	EP + CNT + CF ref	EP with 0.3% CNT	CF	-
5	EP + CNF(CNT) + CF ref	EP	CNF with 2% CNT + CF	-
6	EP + CNF(CNT) + CNT + CF ref	EP with 0.3% CNT	CNF with 2% CNT + CF	-
7	EP FR	EP	-	RDP 2.5% P
8	EP + CNT FR	EP with 0.3% CNT	-	RDP 2.5% P
9	EP + CF FR	EP	CF	RDP 2.5% P
10	EP + CNT + CF FR	EP with 0.3% CNT	CF	RDP 2.5% P
11	EP + CNF(CNT) + CF FR	EP	CNF with 2% CNT + CF	RDP 2.5% P
12	EP + CNF(CNT) + CNT + CF FR	EP with 0.3% CNT	CNF with 2% CNT + CF	RDP 2.5% P

EP: epoxy resin; CF: carbon fiber; CNT: carbon nanotube; CNF(CNT): carbon nanofiber containing carbon nanotubes; FR: flame retardant; RDP: resorcinol bis(diphenylphosphate).

**Table 2:** Fire performance of the prepared reference and flame-retarded samples.

No.	Sample Code	TTI [s]	pHRR [kW/m^2^]	t_pHRR_ [s]	THR/m [MJ/m^2^g]	Residue [wt %]
1	EP ref	22	682	118	2.10	0
2	EP + CNT ref	24	658	170	1.99	0
3	EP + CF ref	44	162	92	0.47	60.0
4	EP + CNT + CF ref	44	174	95	0.49	61.6
5	EP + CNF(CNT) + CF ref	51	163	108	0.49	61.3
6	EP + CNF(CNT) + CNT + CF ref	43	201	117	0.64	54.1
7	EP FR	21	386	103	1.39	0
8	EP + CNT FR	35	390	128	1.35	0
9	EP + CF FR	62	133	106	0.33	57.8
10	EP + CNT + CF FR	55	136	102	0.36	59.0
11	EP + CNF(CNT) + CF FR	58	140	116	0.48	53.5
12	EP + CNF(CNT) + CNT + CF FR	47	130	111	0.43	54.1

TTI: time to ignition; pHRR: peak heat release rate; t_pHRR_: time of peak heat release rate; THR: total heat release; m: mass.

**Table 3:** Limiting oxygen index (LOI) and UL-94 results of the prepared reference and flame-retarded samples.

No.	Sample Code	LOI [*V/V*%]	UL-94
1	EP ref	23	HB (13.9 ± 6.5 mm/min)
2	EP + CNT ref	22	HB (23.6 ± 3.0 mm/min)
3	EP + CF ref	33	HB (1^st^ ignition)
4	EP + CNT + CF ref	30	HB (1^st^ ignition)
5	EP + CNF(CNT) + CF ref	29	HB (1^st^ ignition)
6	EP + CNF(CNT) + CNT + CF ref	30	HB (2^nd^ ignition)
7	EP FR	32	HB *
8	EP + CNT FR	33	HB *
9	EP + CF FR	42	HB (2^nd^ ignition)
10	EP + CNT + CF FR	40	HB (2^nd^ ignition)
11	EP + CNF(CNT) + CF FR	39	V-1
12	EP + CNF(CNT) + CNT + CF FR	39	V-0

* burning time longer than that of V-2.

**Table 4:** Mechanical properties of the prepared reference and flame-retarded samples.

No.	Sample Code	Tensile Strength [MPa]	Young’s Modulus [GPa]	Elongation at Break [%]	Flexural Strength [MPa]	Flexural Modulus [MPa]
1	EP ref	55.5 ± 8.4	2.4 ± 0.5	3.7 ± 2.1	67.7 ± 10.2	2.41 ± 0.5
2	EP + CNT ref	39.8 ± 15.7	3.0 ± 0.4	3.1 ± 3.0	76.9 ± 7.5	2.93 ± 0.4
3	EP + CF ref	452.5 ± 52.9	67.6 ± 12.2	0.5 ± 0.2	635.7 ± 135	62.5 ± 3.72
4	EP + CNT + CF ref	559.2 ± 80.5	99.2 ± 7.2	0.5 ± 0.1	974.5 ± 19.2	71.2 ± 2.57
5	EP + CNF(CNT) + CF ref	452.7 ± 31.5	81.2 ± 15.7	0.5 ± 0.2	502.8 ± 46.5	52.8 ± 9.1
6	EP + CNF(CNT) + CNT + CF ref	444.0 ± 48.3	82.3 ± 6.4	0.5 ± 0.0	841.2 ± 123	71.8 ± 1.4
7	EP FR	6.95 ± 1.3	0.64 ± 0.2	13.9 ± 5.7	3.98 ± 1.9	0.13 ± 0.08
8	EP + CNT FR	4.54 ± 0.2	0.07 ± 0.01	12.8 ± 2.2	1.95 ± 0.02	0.04 ± 0.02
9	EP + CF FR	252.9 ± 28.1	40.2 ± 9.8	0.7 ± 0.0	245.2 ± 37.5	39.1 ± 4.93
10	EP + CNT + CF FR	300.8 ± 73.8	68.1 ± 8.1	0.6 ± 0.2	394.6 ± 26.7	57.0 ± 2.3
11	EP + CNF(CNT) + CF FR	194.4 ± 38.7	29.5 ± 14.9	0.7 ± 0.2	75.0 ± 9.4	5.15 ± 0.7
12	EP + CNF(CNT) + CNT + CF FR	278.4 ± 41.2	41.5 ± 1.9	0.6 ± 0.0	63.1 ± 12.5	4.71 ± 1.7
